# Key Challenges in the Search for Innovative Drug Treatments for Special Populations. Converging Needs in Neonatology, Pediatrics, and Medical Genetics

**DOI:** 10.3390/children4080068

**Published:** 2017-08-04

**Authors:** Stuart MacLeod

**Affiliations:** Faculty of Medicine, University of British Columbia, Department of Pediatrics, British Columbia Children’s Hospital Research Institute, 950 West 28th Avenue, Vancouver, BC V5Z 4H4, Canada; macleod.stuart@gmail.com

**Keywords:** neonatology, pediatrics, rare disorders, clinical trials, clinical pharmacology, pharmacy, innovative therapy, human resources

## Abstract

The explosion of knowledge concerning the interplay of genetic and environmental factors determining pathophysiology and guiding therapeutic choice has altered the landscape in pediatric clinical pharmacology and pharmacy. The need for innovative research methods and design expertise for small clinical trials to be undertaken in sparse populations has been accentuated. At the same time, shortfalls in critical human resources represent a key challenge, especially in low- and middle-income countries where the need for new research and education directions is greatest. Unless a specific action plan is urgently developed, there will be a continuing gap in availability of the essential expertise needed to address treatment challenges in special patient populations such as neonates, patients suffering from rare or neglected diseases, and children of all ages.

## 1. Introduction

For those involved in the interrelated fields of pharmacology, toxicology, and therapeutics during the past half century, there can be no doubt that monumental changes have occurred both in the underlying foundational science that supports clinical choice and in the high priority assigned to safe as well as effective therapeutic outcomes. With the advance of human genomics, the astonishingly rapid progression of biomedical sciences has generated an improved understanding of the interplay between genetic and environmental factors in determining pathophysiology observed in clinical settings. The implications for therapeutic choice are apparent.

Nowhere has the impact of scientific change been more heavily highlighted than in the treatment of special patient populations. Such populations include neonates, patients suffering from rare or neglected diseases, and children of all ages in a variety of settings. 

The opportunity to improve diagnosis and treatment of such unique patient populations has brought new responsibilities to involved clinicians, researchers, and the institutions and organizations with which they are associated. Both opportunities and responsibilities have expanded for those involved in drug discovery and for government regulators.

From the perspective of those committed to improving therapeutics for childhood disorders, many of the forces described may be seen as focused within the disciplines of pediatric clinical pharmacology and pharmacy. Never have the opportunities been greater than at present for pediatric pharmacologists to achieve treatment breakthroughs of lasting benefit for children building on recent progress [1,2,3,4,5,6,7,8]. However, if such breakthroughs are to be achieved, a more carefully orchestrated approach and integration of expertise from diverse researchers and clinicians will be required [9,10,11,12].

## 2. The Need

According to current World Bank statistics (2015), the world’s population of children under the age of 14 is near two billion or about 26% of the total. Such demography would not seem exceptional, but the impact is amplified by the fact that the pediatric population is declining in a majority of the world’s countries that are highly developed economically and biomedically while increasing in poorly resourced settings. In Africa, in particular, the pediatric population is steadily increasing. In Niger, 50% of the population is under the age of 14 and the figure for Angola, Chad, and Uganda is not far behind at 48%. Most other countries in sub-Saharan Africa report populations of children under 14 of between 40% and 46%. In comparison, North American populations under 14 average 19% [13].

Especially in countries with high birthrates, frequently coupled with poor availability of scientific and clinical resources, there will be an accentuated need for expertise in neonatal care and for treatment of older children with life threatening, often neglected, diseases that are commonly coupled with severe malnutrition [14].

Since children in all countries are to some extent deprived of access to well-validated, safe, and effective medicines, child caregivers have become too much resigned to the use of off-label therapies or have resorted to extrapolation of dosage and therapeutic expectations from adult studies. Ito [2] has recently estimated that 50% of drug prescribing in his highly specialized Canadian children’s hospital is off-label. The proportion of off-label treatment escalates dramatically to 80% or higher in neonatal intensive care and other critical care settings [2]. The need for expanded focus on obstetrical and neonatal clinical pharmacology research has been recognized for some time, but relevant human resources are scarce and logistical challenges are abundant [15,16].

A parallel problem is to be found in the management of rare disorders which have increasingly come to occupy a priority position in the therapeutic agenda. While not all rare disorders are pediatric conditions, the majority do present in childhood where the need for supporting therapeutic research is heightened. The needed research agenda accentuates parallels among neonates, children, pregnant or nursing women, and patients with childhood presentations of rare disorders.

It is generally reported that there are at least 7000 potentially treatable rare disorders [17], although this seems likely to be an underestimate. Available knowledge concerning best approaches to diagnosis and management of many rare disorders remains inadequate or unavailable in many treatment centres. Even where accurate genetic diagnosis has become possible, there remain significant gaps in identification and availability of validated therapies. 

With better understanding of epigenetic changes that regulate physiologic and pharmacologic processes, it seems likely that recognition of rare disorders will expand. This is already true in oncology where cancers that were previously thought of as single entities are increasingly being seen as a diverse family of conditions often requiring subtle differentiation in targeting of therapeutic approaches.

An extensive multidisciplinary investigative effort will be required, beginning in pediatrics, in order to fully understand the complex interplay of genetic and environmental factors needed to describe the natural history of rare disorders. Such improved approaches, including innovations such as pre-emptive pharmacogenetic analysis, should lead eventually to the development of novel, safe, and effective therapies [17,18,19,20,21,22].

Confirmation of the importance of this task can be seen in the strategic research agenda of the European Community’s Innovative Medicines Initiative. Under its Innovative Medicines Initiative 2 (IMI2) joint undertaking, proposals are currently under review for research in areas where societal, public health, and biomedical industry competitiveness goals are aligned. This strategic direction directly addresses the need for therapeutic innovation in neonatology, pediatrics and medical genetics through a specific call for creation of a pan-European pediatric clinical trials network [23].

## 3. The Science of Therapeutic Evaluation

It has become clear that differences in response to medicines—both therapeutic and toxic—will commonly have a strong genetic component. Increasingly, clinicians will turn to genome sequencing as a means of identifying clinically important pharmacogenomic variation. Improved therapeutic choice among medicines will inevitably result from better understanding of the role played by genetic diversity in determining response [24].

As biomedical knowledge grows exponentially, the evaluation science required to support regulation of therapeutic products and subsequent clinical choice is continuously evolving. Increased understanding of the human genome is rapidly being translated into an integrative science of pharmacogenomics that will support a rapid shift towards more personalized (precision targeted) therapeutics [25]. Increasingly, studies will be conducted in patient populations that have been genomically characterized with likely beneficiaries of therapy or those at risk of toxicity pre-emptively identified. Such an approach will unquestionably necessitate new research methods, including a radically altered approaches to clinical trials in many situations. The key components of a comprehensive clinical investigation program required for the support of regulatory and clinical decision making in the new environment are outlined in Box 1.

One of the key challenges to be faced will be the need for a closer alignment of health technology assessment with therapeutic evaluation, including the generation of high quality evidence from clinical trials. There are at least five areas that require consideration if improved harmonization is to be achieved [17].

- Evidentiary needs should be more clearly delineated and better aligned to reflect the expectations of regulators, manufacturers, patients/families, and clinical caregivers.
- Early dialogue should be encouraged, bringing together regulatory decision makers with researchers, clinicians, patient/family groups, and sponsors of newly discovered products. Inclusion at this stage of those who will eventually be responsible for reimbursement decisions in public or private domains would also add value.
- Wherever possible, the process should be streamlined so that innovative products are submitted for health technology assessment at the same time as entering regulatory review. Emphasis must be placed as much as possible on the social and clinical context in which the new technology will be applied and not only on the safety and efficacy of the product itself.
- Increasing consideration of some form of adaptive licensing (coverage with evidence development) may facilitate more timely introduction of innovative therapies. The continuing challenge is to align the best possible advice on the design of pre- and post-market evaluations with the expectations of patients, families, and clinicians hoping for early access to promising treatments.
- Especially in the case of rare disorders, it is critically important to engage patients and families at the earliest possible stage of product development. In addition to other obvious benefits, the careful observation of a condition’s natural history and maintenance of detailed registries will greatly facilitate later validated determination of safety and efficacy.

## 4. Institutional Roles and Responsibilities

There are multiple obstacles impeding achievement of a fully integrated approach to the study of therapeutic innovation in unique populations. Success in this complex undertaking requires close collaboration of clinicians, caregiving institutions, patients, and families with multiple research stakeholders, regulatory authorities, and those responsible for the discovery and development of novel therapies from both private and public sectors.

Under current circumstances it can be anticipated that, regardless of where the original breakthrough occurs, most novel therapies will be subsequently developed through private sector efforts and that clinical investigation in phase III and IV will be executed primarily in tertiary and quaternary care hospitals, usually equipped for academic coordination of clinical care and research.

Unfortunately, as described below, the human resource complement available for support of such vital research is limited and this is particularly true in the case of research required to serve the needs of special and unique populations. As a generalization, it can be observed that significant funding has been available over at least the past two decades for support of basic research in pathophysiology, pharmacology, toxicology, genetics, and epidemiology. Nonetheless, the key underpinnings of therapeutic investigation in most countries, including those with the highest levels of biomedical development, investment has lagged in clinical research infrastructure and in the support for training of key personnel needed to implement an increasingly challenging research strategy.

There are approximately 2000 accredited medical schools in the world [26], but only a limited proportion of those schools have a comprehensive research capacity including clinical research facilities and expertise necessary to address therapeutic evaluation needs in special populations. A dramatic increase in availability of both fiscal and human resources will be required if the improved clinical treatment opportunities presented by recent scientific breakthroughs are to be realized across the diverse range of economic settings.

As noted previously with respect to the distribution of the world’s children, a similar pattern prevails in clinical research expertise. Most of the necessary capacity is found in highly sophisticated and specialized hospitals located in countries with the highest levels of economic development. This maldistribution of infrastructure will continue to undermine efforts to bring modern innovative therapies to most of the world’s population unless an international integrated action plan is agreed and implemented [11,12].

## 5. Human Resources

Institutions face major obstacles in meeting their responsibilities with respect to therapeutic needs of special populations. The institutional dilemma is reinforced under current political and research funding models. So far, governments have, for the most part, failed to accept a responsibility to ensure the availability of innovative and validated therapy for patients with unique needs.

Because the problem has gone unacknowledged, investment in training of key personnel who would be appropriately qualified to address the crisis has been limited. Two recent publications have addressed the worldwide shortfall in individuals qualified to study medicines in children [11,12]. While these studies focused on pediatrics, they did indicate an even more alarming shortfall in neonatology. Were the same questions to be posed about human resource availability for study of innovative rare disorder treatments, results would undoubtedly be similar.

In the case of rare disorders, an immensely powerful scientific engine has been created that is capable of characterizing genetic conditions at an astonishing rate. To date, however, there has been no adequate parallel program devoted to the training of clinical research personnel who will be available to validate a host of novel therapies soon to be forthcoming.

## 6. Conclusions

It is apparent that, in the ongoing search for innovative drug treatments, the clinical research needs in pediatrics, neonatology, and clinical genetics converge. Unless a specific action plan is soon agreed upon, there will be a continuing gap in availability of essential expertise to address the unique research requirements described. While this commentary is written from the perspective of a clinical pharmacologist, the world’s clinical research needs will encompass many disciplines as indicated in Box 1.

As concluded in a recent parallel publication, and paraphrased here: “A process of targeted strategic planning should be undertaken with potential supporters of clinical training programs to heighten the focus on clinical investigation in areas of priority concern, such as neonatology, mental health, neurology, genetics/rare disorders, communicable disease and oncology” [12].

Box 1 **Key components: improved clinical studies of innovative therapies for special populations.**

Design expertise for small clinical trials undertaken in sparse populations (alternative and adaptive designs) [27]Tools for dose finding and standardizationInnovative statistical methods/Bayesian and other non-frequentis approachesModeling/simulation capability to support protocol developmentAnalytic capacity (including measurement of biological entities)Pharmacokinetics/pharmacodynamicsClinical toxicology [28]Pharmacogenetics/pharmacogenomicsRisk management/data safety monitoringData transfer/data management/use of clinical registries in trialsCapability of formulation adaptation to special populations [29]Implementation science/alignment of health technology assessment with therapeutic evaluationImproved knowledge dissemination strategies
